# Manipulation of magnetization switching and tunnel magnetoresistance via temperature and voltage control

**DOI:** 10.1038/srep18269

**Published:** 2015-12-14

**Authors:** Houfang Liu, Ran Wang, Peng Guo, Zhenchao Wen, Jiafeng Feng, Hongxiang Wei, Xiufeng Han, Yang Ji, Shufeng Zhang

**Affiliations:** 1 Beijing National Laboratory of Condensed Matter Physics, Institute of Physics, Chinese Academy of Sciences, Beijing 100190, China; 2 SKLSM, Institute of Semiconductors, Chinese Academy of Sciences, Beijing 100083, China; 3 Department of Physics, University of Arizona, Tucson, Arizona 85721, USA

## Abstract

Magnetization switching between parallel and antiparallel alignments of two magnetic layers in magnetic tunnel junctions (MTJs) is conventionally controlled either by an external magnetic field or by an electric current. Here, we report that the manipulation of magnetization switching and tunnel magnetoresistance (TMR) in perpendicularly magnetized CoFeB/MgO/CoFeB MTJs can be achieved by both temperature and voltage. At a certain range of temperature, coercivity crossover between top and bottom magnetic layers is observed in which the TMR ratio of the MTJs is almost unmeasurable. Furthermore, the temperature range can be tuned reversibly by an electric voltage. Magnetization switching driven by the voltage reveals an unconventional phenomenon such that the voltage driven coercivity changes with temperature are quite different for top and bottom CoFeB layers. A model based on thermally-assisted domain nucleation and propagation is developed to explain the frequency and temperature dependence of coercivity. The present results of controlling the magnetization switching by temperature and voltage may provide an alternative route for novel applications of MTJs based spintronic devices.

Magnetic tunnel junctions (MTJs), comprised of a tunnel barrier sandwiched between two ferromagnetic layers, have emerged as essential building blocks for magnetic random access memories (MRAMs) and spin logic devices^1^,^2^,^3^,^4^,^5^,^6^. A crucial issue for reliably operating MTJ based magnetic devices is to find most efficient ways to switch two magnetic layers from parallel (P) to antiparallel (AP) (and vice versa) alignments of the magnetization. At present, a common scheme is to utilize an electric current that either generates a large magnetic field or creates a strong spin transfer torque for controlling the magnetization^7^,^8^,^9^,^10^. The current density required for the current-driven switching remains to be prohibitively large and energetically expensive. Most recently, a voltage-assisted magnetization switching has been successfully demonstrated^11^,^12^,^13^. In this method, voltage applied to the interface of a magnetic metal and an insulator induces a significant charge transfer at the magnetic interface^14^,^15^,^16^,^17^,^18^. Through strong interface spin-orbit coupling, these transferred charges result in a change of the interface perpendicular magnetic anisotropy (PMA). Indeed, the voltage-controlled PMA has successfully applied to MTJs where the PMA in both magnetic layers are tunable and only a very small current density is needed to switch the magnetization, which is extremely encouraging in terms of reducing the power consumption for spintronic devices^11^.

Here, we report novel switching characteristics in designed Co_40_Fe_40_B_20_/MgO/Co_40_Fe_40_B_20_ perpendicular magnetic tunnel junctions (p-MTJs) when we vary temperature and voltage. The top and bottom magnetic layers usually have different temperature dependence of the coercivity. It is found that the crossover in which coercivity of the two layers becomes equal occurs at certain temperatures whose range can be tuned reversibly by an electric voltage. One consequence of the coercivity crossover is the disappearance of TMR in which the anti-parallel alignment of the two layers cannot be formed during the magnetic field sweeping. Another interesting observation is that voltage-induced coercivity change shows different characteristics with temperature for the top and bottom CoFeB layers: at high temperature the coercivities of the top and bottom CoFeB layers have an opposite dependence on the electric voltage, exhibiting quasi-linear function; while at low temperature an even function voltage dependence for top CoFeB layer is observed but almost no change in bottom CoFeB layer. Thus the previously recognized model of the voltage induced anisotropy change based on charge transfer at interfaces may not be applicable to our findings. By using several different methods presented in detail below, we have found that the strong dependence of the coercivity on temperature and voltage is dominated by domain nucleation and propagation, and to a less extent, by the perpendicular anisotropy change. A semi-quantitative model of thermal-assisted magnetization reversal qualitatively accounts for the experimental data on the frequency and temperature dependence of the coercive fields of both top and bottom magnetic layers.

Experiments were carried out on sputter-deposited films with layered structure of substrate/Ta(5)/Ru(10)/Ta(5)/CoFeB(1.2)/MgO(2)/CoFeB(1.4)/Ta(5)/Ru(6) where the numbers in parenthesis are thickness in the unit of nanometers. The films were subsequently patterned into rectangular pillars with the lateral size of 5 × 10 μm^2^ for magneto-electrical transport measurement. Two other structures with a single magnetic layer consisting of substrate/Ta(5)/MgO(2)/CoFeB(1.4)/Ta(5) in which CoFeB is on the top of MgO layer (t-CoFeB) and substrate/Ta(5)/CoFeB(1.2)/MgO(2) /Ta(5) with CoFeB on the bottom of MgO (b-CoFeB) are fabricated for magnetic property studies. Details of sample preparation are presented in the Methods. In those structures, a proper thickness of CoFeB layers is crucial since PMA has only been found within the thickness range between 1.2 nm and 1.5 nm^19^.

In Fig. 1a, the TMR ratios as a function of external magnetic field are shown at a low temperature (2 K) and at room temperature (RT). The squared hysteresis loops for the applied perpendicular magnetic field indicate the perfectly perpendicular easy axis for both top and bottom magnetic layers. A large TMR ratio of 293% at 2 K (128% at RT) was achieved, indicating the high quality of the p-MTJs (Supplementary Section 1). The bias field arising from dipolar interlayer coupling is about 5 Oe at both RT and 2 K, much less than that reported by H. D. Gan *et al.*^20^. A sharp switching between P and AP configurations is achieved, with the switching fields of 47 Oe and 105 Oe for the top and bottom CoFeB layers at RT. The out-of-plane *M-H* loop also shows two clear steps, which indicate the magnetization switching of top and bottom CoFeB layers. From the hard axis hysteresis measurement, we determined the effective perpendicular anisotropy is 3.1 × 10^6^ erg/cm^3^ for Ta/CoFeB (1.2)/MgO and 5.4 × 10^6^ erg/cm^3^ for Ta/MgO/CoFeB (1.4) at RT. Figure 1b shows differential tunnel conductance dI/dV in P configuration at 2 K. The zero field anomaly as well as the two shoulders are typical of spin dependent tunneling through MgO barrier^21^,^22^,^23^. Comparing the above transport and magnetic properties with previous measurement by other groups confirms that our samples are among the highest quality^11^,^18^,^24^,^25^,^26^. The temperature dependence of parallel (*R*_*P*_) and antiparallel (*R*_*AP*_) resistance and TMR ratio, as shown in Fig. 1c, exhibits typical tunnel characteristics. The TMR ratio gradually decreases from low temperature to room temperature. The resistance under a large magnetic field where the magnetic layers are parallel is nearly temperature independent, and thus the temperature dependence of the TMR is mainly from the decrease of the resistance of the AP configuration as the temperature increases. An anomaly, however, occurs at a narrow range of temperature between 140 K and 145 K where the TMR could not be measured (Supplementary Section 2). By inspecting the magnitude of the resistance during the magnetic field sweeping, we find that the AP configuration does not have noticeable resistance jumps. Thus, we ascribe the anomaly between 140 K and 145 K to the fact that the coercivity difference of two magnetic layers is vanishing such that two layers cannot form the antiparallel state.

Figure 2a shows the temperature dependence of coercive fields of the top and bottom CoFeB layers from the *R*(*H*) loops of the p-MTJ. The magnetization switching of top and bottom CoFeB layers can be determined by anomalous Hall measurement in a separated top or bottom CoFeB structure (Supplementary Section 3). The coercive field is calculated from the average of positive and negative switching fields 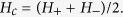 Clearly, the coercivity of the bottom CoFeB layer is much less temperature dependence than that of the top CoFeB layer; this is consistent with the general picture of the growth dynamics: a metal grown on an insulator tends to have a rougher interface than that of an insulator grown on a metal^27^. Thus, the top CoFeB layer would have a wider distribution of the nucleation energy and a sharper temperature dependence of the coercivity. At low temperature, the coercivity of top CoFeB layer is much larger than that of bottom CoFeB layer. At around 145 K, the coercivity becomes comparable. The disappearance of TMR ratio at this temperature indicates that the switching of the two layers occurs simultaneously and the AP state is unstable. When the temperature further increases, however, the coercivity of bottom CoFeB layer becomes larger than that of top CoFeB resulting in the TMR re-appears, as shown in Fig. 1(c). The magnetic properties of the unpatterned p-MTJ film were measured at different temperatures, which are shown in Fig. 2b. The inset of the Fig. 2b shows the *M* (*H*) loops from 10 K to 300 K. A similar anomaly as in Fig. 2a occurs at a narrow range of temperature between 175 K and 200 K, which is a little higher than that of the patterned p-MTJ. This is probably related to the micro-size effect. Previously, H. D. Gan *et al.* also found that the collapse of TMR of 40 nm diameter CoFeB/MgO/CoFeB p-MTJs after annealed at high temperature, but they attributed the mechanism to an induced magnetic coupling between two layers (which makes independent rotation of the magnetization of the two layers impossible) rather than the temperature dependent coercivity^20^.

In order to understand the origin of the temperature dependence of the top and bottom layers, we have separately grown unpatterned t-CoFeB and b-CoFeB films whose thickness and growth conditions are identical to those in the MTJ. An alternate magnetic field, 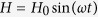, was applied perpendicularly to the plane of the films, where *H*_0_ is the amplitude of applied magnetic field, ***ω*** is the frequency of magnetic field, and *t* is measuring time. The dynamic coercivity, defined as the instantaneous field at which the magnetization jumps from negative to positive values, is measured by a MOKE (magneto-optical Kerr effect) system with a 632.8 nm laser beam spot of diameter in tens of microns (See Method). In this time-dependent measurement, the jumps occur at different fields for each cycle of the applied ac magnetic field, and thus the dynamic coercivity takes as an average of at least 10 hysteresis cycles. Figure 3a,b show the dynamic coercivities of t-CoFeB and b-CoFeB films as functions of the alternate magnetic field amplitude *H*_0_ at a fixed frequency of 50 Hz and magnetic field frequency *f* at fixed amplitude of 200 Oe with the temperature of 100, 200, and 300 K, respectively. The strong dependence of the dynamic coercivity on the temperature as well as the amplitude of the ac magnetic field indicates a significant role of thermally assisted magnetization switching for both top and bottom CoFeB layers. To understand these data quantitatively, we introduce a thermal-assisted model based on domain nucleation and expansion under a reversing magnetic field^28^,^29^. When a constant reversing magnetic field is applied, the coercivity can be readily derived from the model^30^,


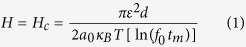


where 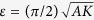 is the domain wall energy per area with *A* exchange stiffness and *K* anisotropy constant;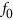, *T* and 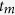 are the attempt frequency, the temperature and the experimental measuring time, respectively; *d, a*_0_ and *κ*_B_ are the thickness of the film, the unit cell length (lattice constant for simple cubic structure) and Boltzmann constant, respectively. The fitting experimental curves and parameters are shown in Fig. S5 and table 1 of Supplementary Section 4, respectively. For b-CoFeB, the domain wall energy 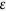 decreases from 0.94 × 10^−2^ J/m^2^ at 100 K to 0.47 × 10^−2^ J/m^2^ at 300 K, while for the t-CoFeB, the domain wall energy 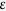 firstly decreases from 1.25 × 10^−2^ J/m^2^ at 100 K to 0.92 × 10^−2^ J/m^2^ at 200 K, and then suddenly increases up to 1.33 × 10^−2^ J/m^2^ at 300 K. The temperature dependence of the domain wall energy 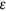 is not strong (within the same order of magnitude), but sizable. While Eq. (1) predicts that the coercivity is inversely proportional to the temperature, one should be cautious that the domain wall energy 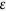 usually decreases with the temperature increase because of the reduced exchange and the magnetic anisotropy. Thus, the temperature dependence of the coercivity is usually weaker than the inverse relation with the temperature. Another point is that our model becomes less robust when the temperature is high and the pinning potential becomes weak. In this case, other coercive mechanisms such as the coherent rotation and edge domain nucleation may dominate. As in the case of the bottom ferromagnetic layer, the interface is rather smooth and pinning is weak, and thus the coercivity of the bottom layer depends weakly on the temperature and the measurement time (See Supplementary Section 4 for more details).

It is known that the coercivity of the two magnetic layers at RT can also be altered by an applied voltage across the p-MTJs^11^,^31^ through the electric-field-induce perpendicular anisotropy change. Since the anisotropy is proportional to the coercivity for the coherent rotation of the magnetization reversal^13^, the voltage would directly affect the relative coercivity, i.e., the voltage at a fixed bias direction is to enhance the coercivity for one layer and to suppress the coercivity for the other layer. In our p-MTJs, however, the coercivity is originated from the thermal assisted processes, which are weakly dependent on the strength of the perpendicular anisotropy in contrast with coherent rotation magnetization solely determined by the magnetic anisotropy field 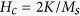. Other parameters such as the temperature and pinning potential also play critical roles in the coercivity. A small voltage-induced anisotropy change may not be important for the observed significant coercivity reduction. Thus it has been unclear whether and how the coercivity is affected by the voltage. In Fig. 4a, we show the voltage induced switching of the magnetization of the two magnetic layers at 100 K: we initialized the MTJs in a anti-parallel state by applying a large magnetic field, followed by applying a reverse magnetic field of *H*_bias_ = 250 Oe, so that the bottom CoFeB layer whose *H*_*c*_ = 210 Oe is switched to the direction parallel to the applied field while the top CoFeB whose *H*_*c*_ = 275 Oe remains in the initial direction, creating the anti-parallel state. A bias voltage *V*_bias_ is then swept from 0 V to ±0.4 V. A distinct resistance jump occurs at the critical bias voltages of −0.13 V and 0.34 V, indicating the magnetization switching from the AP to the P state. Then a reverse magnetic field smaller than the coercivity of the either layer is also applied, i.e., the reverse magnetic field does not induce the switching of either layer. However, the magnetization switching from P to AP state is not observed for the modest magnitude of the voltage. These results indicate that the voltage dependence of the coercivity for the top and bottom CoFeB layers at *T* = 100 K is quite different. This phenomenon on the different voltage dependence is not uncommon in the magnetic thin films. It remains an open question on the physical mechanism of the voltage driven coercivity change. Moreover, since the current density is very low, about 2 × 10^3^ A/cm^2^, the effect of spin transfer torque can be ignored.

An interesting question arises on the sign of the bias voltage. Previous results showed the voltage decreases anisotropy in one polarity but increases anisotropy in another polarity in single MgO barrier p-MTJs. In *Applied Physics Express* **6** (2013) 073005, the authors observed that voltage bias for both polarities always enhances the perpendicular anisotropy of the Fe_80_B_20_ layer (corresponding to our top layer) at RT, while we have observed a decrease in coercivity. This seemingly contradictive result implies the very different role of voltage on the anisotropy and on the coercivity. In the above referenced paper, an in-plane Fe layer is served as the bottom layer of the MTJ junction and an in-plane magnetic field (competing with the top layer anisotropy) determines the angle between two magnetic layers, and thus the perpendicular anisotropy is measured in the experiment. It is noted that the perpendicular anisotropy is only changed by a few percentage by the voltage. In our case, the bottom layer is also a perpendicular layer and the magnetic field is applied perpendicularly. Thus, we measure the dependence of the voltage on the coercivity. Therefore, it is not surprising that we have observed the opposite voltage dependence and we have found a much large percentage voltage-driven change of coercivity, even though both results show an even dependence on the voltage.

Moreover, we can rule out the heating effect because 1) the temperature increases at this voltage is small (Supplementary Section 5), 2) the critical switching voltage is highly asymmetric at positive and negative bias (−0.13 V and 0.34 V), and 3) this bias polarity asymmetry can also be seen by measuring the critical temperature where the coercivity of the two layers is equal: for *V*_bias_ = 0.4 V, it is *T* = 125 K while for *V*_bias_ = −0.4 V, it is 150 K, as shown in Fig. 4b. While the microscopic origin of the voltage-controlled coercivity for the non-uniform rotation magnetization reversal is unclear at present, one possibility is that the effect of the voltage is to reduce the domain wall pinning potential, rather than to alter the perpendicular anisotropy. In our thermal-assisted model, the random distribution of the pinning potential would be enhanced and reduced by the voltage. The magnetization reversal always takes place from the weakest energy barrier, and thus the voltage in either polarity can create a weaker potential and trigger the magnetization reversal, i.e., the applied voltage reduces the coercivity in either bias polarity.

To further understand the mechanism, the coercivities of the top and bottom CoFeB are tuned by bias voltage at various temperatures. The coercivities of the top and bottom CoFeB layers in the p-MTJ as a function of *V*_bias_ at 50 K are shown in Fig. 4c. In comparison with the bottom CoFeB layer, the coercivity of the top CoFeB layer depends strongly on the applied voltage and decreases in either bias polarity, due to the rougher interface and wider distribution of nucleation energy in top CoFeB layer. As we increases the temperature to 300 K, the effect of voltage on coercivity displays a normal bias dependence, i.e., the bottom coercivity increases for the positive bias but decreases for the negative bias voltage, as shown in Fig. 4d. We postulate that the different voltage dependence at low and high temperature is correlated with the different coercive mechanisms: at temperatures below 200 K, the magnetization reversal is dominated by the domain wall nucleation and propagation, while the mixture of the coherent rotation and domain wall motion may coexist at higher temperatures. Our earlier ac field dependent measurement has supported this view point since the thermal assisted model fits our experimental data much better at low temperatures than at high temperatures, especially for top CoFeB layer.

Finally, we illustrate the temperature and voltage dependence of the coercive fields by mapping out the entire range of temperature from 1 K to 300 K and of voltage from −1.0 V to 1.0 V. In Fig. 5a,b, we show the coercivity landscape for the top and bottom CoFeB layers by measuring a series of *R*-*H* loops under different bias voltage *V*_bias_ and temperature *T*, while the TMR ratio landscape as function of *V*_bias_ and *T* is shown in Fig. 5c. Three distinct regions are identified: a) the coercivity of the top CoFeB layer is smaller than that of the bottom CoFeB at high temperatures (T > 200 K), b) the coercivity of the top CoFeB layer is larger at low temperature (T < 100 K), and c) the crossover regime where the relative coercivity of the two layers can be tuned by either temperature or voltage. The equal-coercivity of top and bottom CoFeB layers exists in the region with a dotted line where the disappeared TMR ratio can be achieved due to the difficulty in achieving an anti-parallel state.

In summary, we show that the manipulated magnetization switching and tunnel magnetoresistance via temperature and voltage. The coercivity of top and bottom CoFeB layers in the p-MTJs displays different temperature and voltage dependence. By properly tuning the temperature and voltage, the p-MTJ can be used for various applications such as temperature sensors and voltage-controlled MRAMs. The physical origins of the temperature and voltage dependence of the coercivity could be dominated by the domain nucleation and domain wall propagation through thermal activation. Further study is still needed to elucidate the microscopic origin of the voltage controlled magnetization switching.

## Method

The multilayers of Ta(5)/Ru(10)/Ta(5)/CoFeB(1.2)/MgO(2)/CoFeB(1.4)/Ta(5)/Ru(6), Ta(5)/MgO(2)/CoFeB(1.4)/Ta(5) (t-CoFeB) and Ta(5)/CoFeB(1.2)/MgO(2)/Ta(5) (b-CoFeB) (all thicknesses in nm) were deposited on oxidized silicon wafer by the sputtering system. The CoFeB denotes Co_40_Fe_40_B_20_ alloy. Magnetic tunnel junctions with a cross section of 5 × 10 μm^2^ are fabricated by UV-lithography and Ar-ion milling, and then are annealed at different temperatures in vacuum with perpendicular magnetic field of about 8000 Oe for 1 hour at an optimal annealing temperature. The magnetic properties of thin film are measured by vibrating sample magnetometer (VSM) and superconducting quantum interference device (SQUID). The cross-sectional high resolution transmission electron microscopy (HRTEM) is also carried out to check the quality of MgO barrier. The electro-magnetic transport properties are measured in a physical properties measurement system (PPMS) with a four-probe measuring technique.

The dynamic hysteresis loops of t-CoFeB and b-CoFeB MTJs are measured by a MOKE (magneto-optical Kerr effect) system setup with a 632.8 nm laser beam spot of diameter in tens of microns. The magnetic field detected by Hall probe with frequency response ranges from 2 Hz to 50 kHz is produced via an electromagnet driven by an alternating current at a frequency up to 400 Hz. The frequency of ac power source can be continuously adjustable from 50 Hz to 400 Hz, and voltage amplitude can be up to 300 V during the frequency range. The inductance of electromagnet coil is about 90 mH. With these parameters, we are able to achieve the amplitude of the ac field more than 1 kOe at 50 Hz. To make the hysteresis loops contain enough points without distortion, a data acquisition (DAQ) card with the acquisition bandwidth up to 2 MHz per channel is used to record periodic magnetic field *H* and Kerr signal *M* simultaneously. Using liquid helium as the cryogen, the temperature of the samples in a vacuum cavity can be tuned from 7 to 300 K. From low to high temperatures, the hysteresis loops are collected by two sets of procedures: varying the voltage applied to the electromagnet with a gradually increasing amplitude of the external field at a constant frequency, or with a gradually increasing frequency of the external field at a fixed amplitude. Each MOKE signals for dynamics coercivity are averaged by at least 10 cycles.

## Additional Information

**How to cite this article**: Liu, H. *et al.* Manipulation of magnetization switching and tunnel magnetoresistance via temperature and voltage control. *Sci. Rep.* **5**, 18269; doi: 10.1038/srep18269 (2015).

## Supplementary Material

Supplementary Information

## Figures and Tables

**Figure 1:**
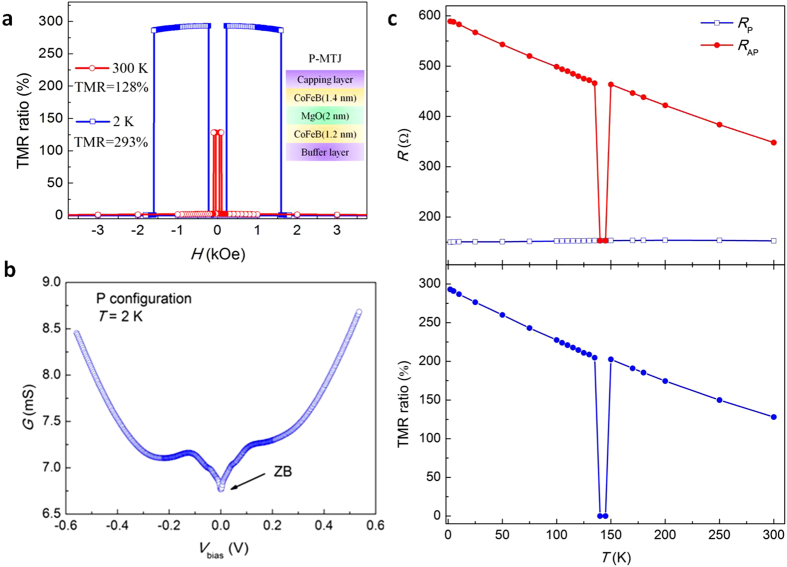
Magnetic transport properties of a CoFeB/MgO/CoFeB p-MTJ. (**a**) TMR ratio as a function of out-of-plane magnetic field at 2 K and 300 K, respectively. (**b**) The dynamic tunneling conductance at 2 K for parallel configuration of magnetization. (**c**) *R*_*P*_, *R*_*AP*_ and TMR ratio of the CoFeB/MgO p-MTJ as a function of measuring temperature.

**Figure 2:**
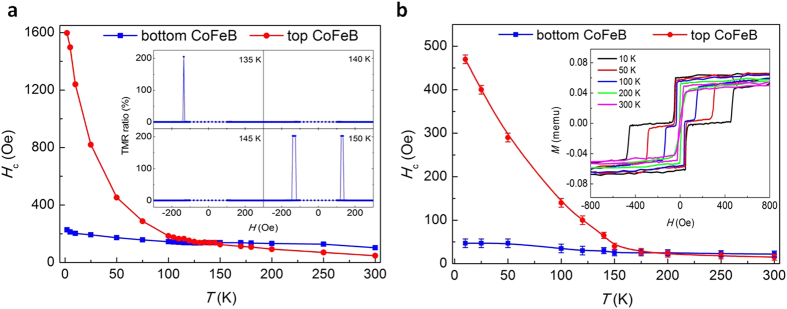
Temperature dependence of coercivity derived from *R*(*H*) and *M*(*H*) loops. (**a**) Temperature dependence of coercivity of the top and bottom CoFeB layers, obtained from the *R*(*H*) loops of the p-MTJ. The inset shows out-of-plane TMR ratio with field scanning in the temperature region from 135 K to 150 K. (**b**) Temperature dependence of coercivity of the top and bottom CoFeB from unpatterned p-MTJ films. The inset shows *M*-*H* loops at different temperatures.

**Figure 3:**
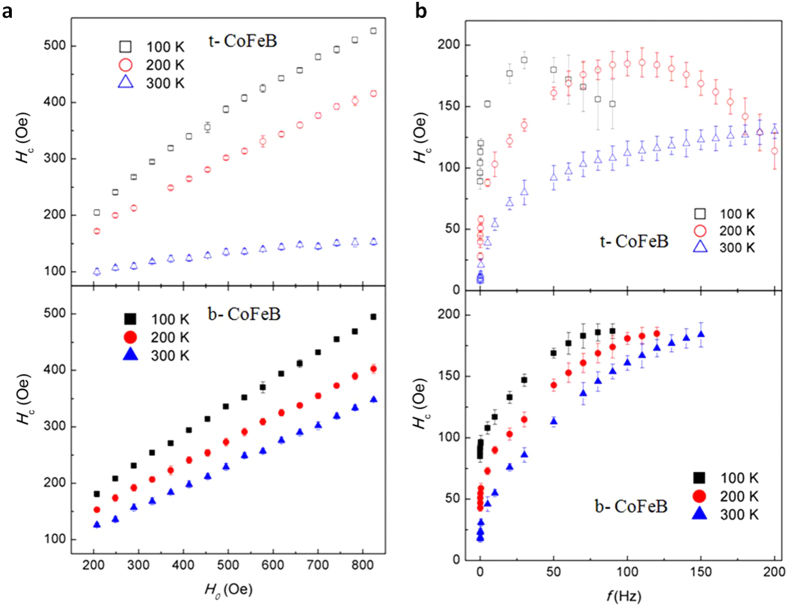
Dynamic coercivity as a function of magnetic field with varying amplitude and frequency. (**a**) Dynamic coercivities of t-CoFeB and b-CoFeB layers at a fixed frequency of 50 Hz. (**b**) Dynamic coercivities at a fixed amplitude of *H*_*0*_ = 200 Oe. The external magnetic field is applied perpendicular to the film plane. The dynamic coercivities are extracted from a series of dynamic hysteresis loops of t-CoFeB and b-CoFeB films, which are measured at the temperature of 100, 200 and 300 K by a MOKE system.

**Figure 4:**
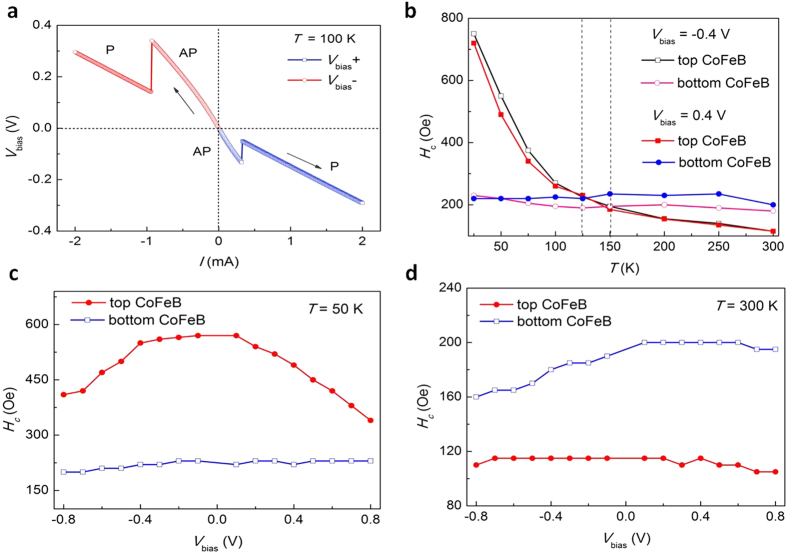
Electric voltage assisted magnetization switching. (**a**) Typical *I*-*V* curves for the CoFeB/MgO/CoFeB p-MTJ in which the applied voltage in either polarity can switch the magnetization from antiparallel to parallel state. The MTJ was initially set in an antiparallel state with a magnetic field of *H*_bias_ = 250 Oe. (**b**) The coercivity of top and bottom CoFeB in the p-MTJ as a function of temperature at *V*_bias_ =  ±0.4 V. The coercivity crossover points were observed at the temperature of 125 K and 150 K for *V*_bias_ = 0.4 V and −0.4 V, respectively. (**c**,**d**) The coercivity of top and bottom CoFeB layers in the p-MTJ as a function of *V*_bias_ at 50 K and 300 K, respectively.

**Figure 5:**
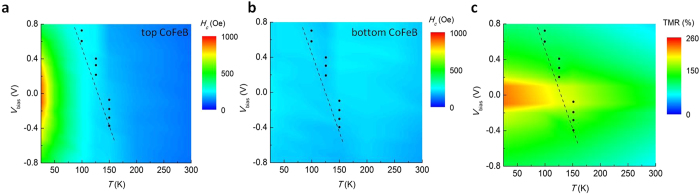
The coercivity and TMR landscape under different bias voltage and temperature. (**a,b**) The coercivity landscape for the top and bottom CoFeB layers by measuring a series of *R*-*H* loops under different bias voltage *V*_bias_ and temperature *T*. Blue solid circles correspond the coercivity crossover points, indicating that the switching of the two layers occurs simultaneously and the AP state is unstable. (**c**) TMR ratio for the p-MTJ summarized from *R*-*H* loops under different bias voltage *V*_bias_ and temperature *T*. Blue solid circles correspond the points where the TMR ratio nearly vanishes. The dash lines are guides for the eyes.
