# Supplementary material for: Manipulation of magnetization switching and tunnel magnetoresistance via temperature and voltage control
Source: Sci Rep. 2015 Dec 14;5:18269. doi: 10.1038/srep18269 (PMC4677325; doi:10.1038/srep18269)
Supplement: Supplementary Information [file srep18269-s1.doc]

Supplementary Information

**Manipulation of magnetization switching and tunnel magnetoresistance via temperature and voltage control**

Houfang Liu^1^, Ran Wang^2^, Peng Guo^1^, Zhenchao Wen^1^, Jiafeng Feng^1^, Hongxiang Wei^1^, Xiufeng Han^1★^, Yang Ji^2^ and Shufeng Zhang^3★^

^1^Beijing National Laboratory of Condensed Matter Physics, Institute of Physics, Chinese Academy of Sciences, Beijing 100190, China, ^2^SKLSM, Institute of Semiconductors, Chinese Academy of Sciences, Beijing 100083, China, ^3^Department of Physics, University of Arizona, Tucson, Arizona 85721, USA.

*E-mail: [xfhan@iphy.ac.cn](mailto:xfhan@iphy.ac.cn), [zhangshu@email.arizona.edu](mailto:zhangshu@email.arizona.edu)

**S1. Structure analysis of CoFeB/MgO/CoFeB p-MTJs**

Fig. S1 shows the cross-sectional TEM images of the p-MTJ made of Ta(5)/Ru(10)/Ta(5)/ Co_40_Fe_40_B_20_(1.2)/MgO(2)/ Co_40_Fe_40_B_20_(1.4)/Ta(5)/Ru(6) (thickness in nm) film in (a) as-grown state and (b) annealed at 300 ºC in vacuum with the magnetic field of up to 8000 Oe. Note that the morphology of the MTJs is smooth the interfaces between the CoFeB electrodes and the MgO barrier are sharp and clearly identifiable, after been annealed at 300 ºC for an hour. The annealing protocol yields high TMR ratios.


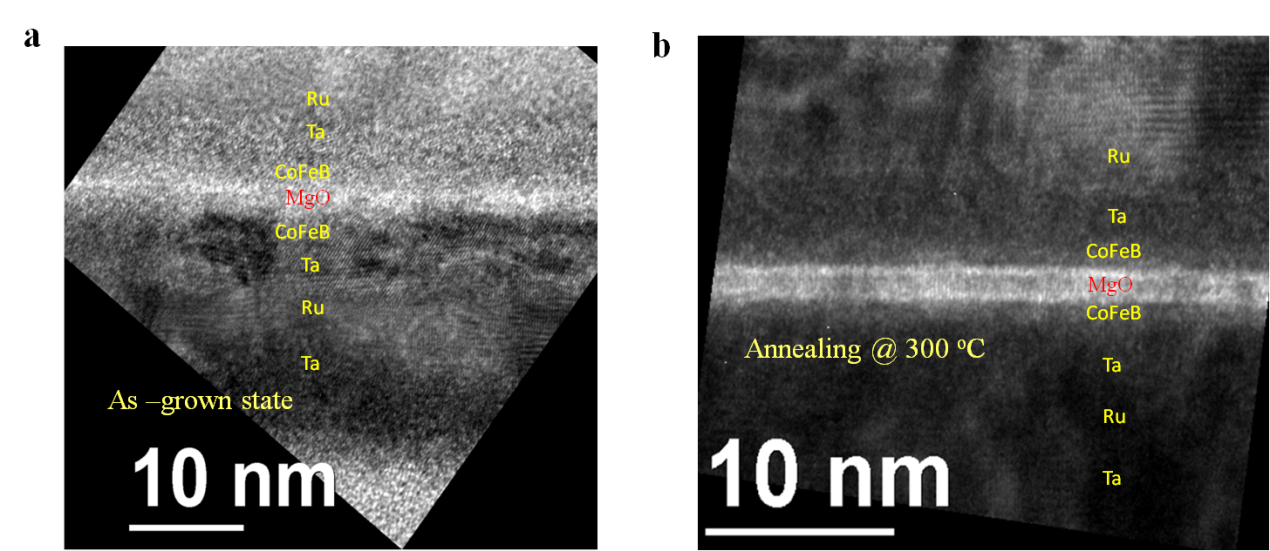


Figure S1. The cross-sectional TEM images of the p-MTJ with the structure of Ta(5)/Ru(10) /Ta(5)/ Co_40_Fe_40_B_20_(1.2)/MgO(2)/Co_40_Fe_40_B_20_(1.4)/Ta(5)/Ru(6) (thickness in nm) film in (a) as-grown state and (b) annealed at 300 ºC in vacuum with the magnetic field of up to 8000 Oe.

**S2. TMR curves for CoFeB/MgO/CoFeB p-MTJ at different temperatures**

Fig. S2 shows the TMR curves in the temperature range from 140 K to 145 K. The TMR ratio of the MTJ is reduced to an extremely small value at 1.5%, which indicates the two layers are not exactly parallel although the coercivity of the top and bottom CoFeB are considered equal-value.


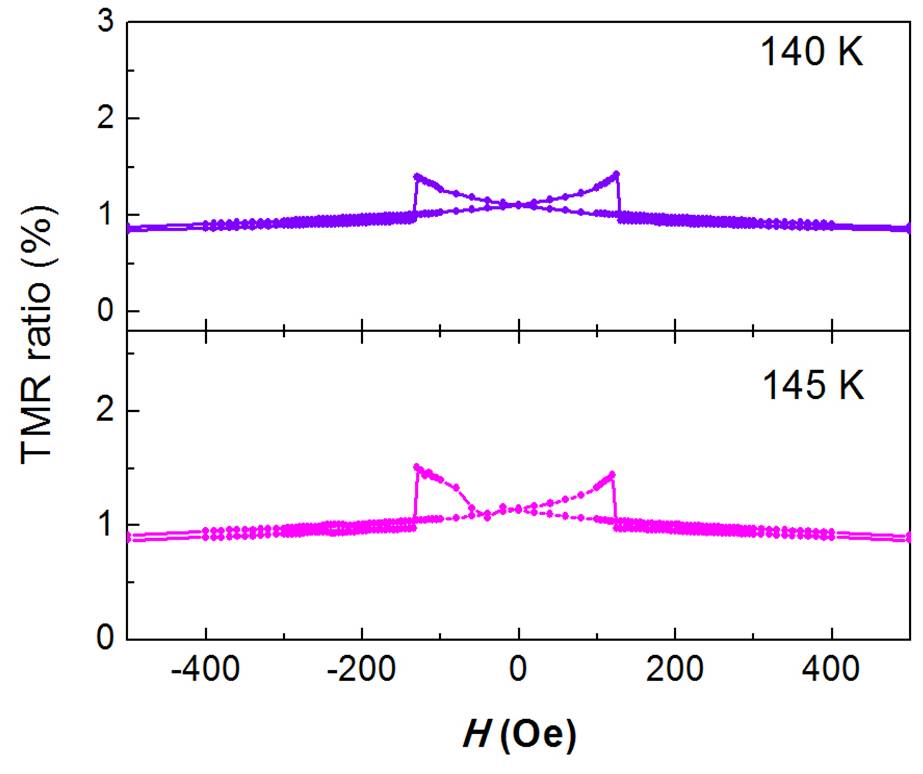


Figure S2. TMR ratio as a function of out-of-plane magnetic field at the temperature of 140 K and 150 K.

**S3. Measurement of anomalous Hall effect with single CoFeB layer**

The coercivity of bilayer CoFeB/MgO (top or bottom CoFeB) extended films is measured by using the anomalous Hall effect (AHE). The Hall resistance *R*_Hall_ can be expressed as,

$R_{Hall}=\frac{R_{0}}{t}H+\frac{R_{e}}{t}M_{\perp}$ (1)

The first term on the right-hand side is the ordinary Hall resistance and the second term is the anomalous Hall resistance which is proportional to the out-of-plane component of the total magnetization ^1, 2^.

The multilayers of top Ta/MgO(2)/CoFeB (1.2 nm)/Ta (t-CoFeB) and bottom Ta/CoFeB(1.4 nm)/MgO(2)/Ta (b-CoFeB) were prepared on thermally oxidized silicon substrate and subsequently were annealed at 300ºC. As seen from Fig. S3(a), the coercivity *H_c_* of t-CoFeB and b-CoFeB are 15 Oe and 85 Oe at room temperature (RT), respectively. The different coercivity is consistent with the general growth trend that the insulator grown on a metal leads to a rougher interface compared to the metal grown on the insulator. Figure S3(b) shows the coercivity *H_c_* for t-CoFeB and b-CoFeB from RT to 5 K. The coercivity crossover has been observed in the temperature range from 140 K to 145 K, which is in consistent with the results seen from *R*-*H* loops of the p-MTJ in the main text. The coercivity *H_c_* of t-CoFeB increases dramatically from 15 Oe at RT to 745 Oe at 5 K, while the coercivity *H_c_* of b-CoFeB only increases slightly from 85 Oe at RT to 180 Oe at 5 K. The different temperature dependence of magnetic properties of t-CoFeB and b-CoFeB indicates a much wider distribution of pinning potentials for the t-CoFeB films.


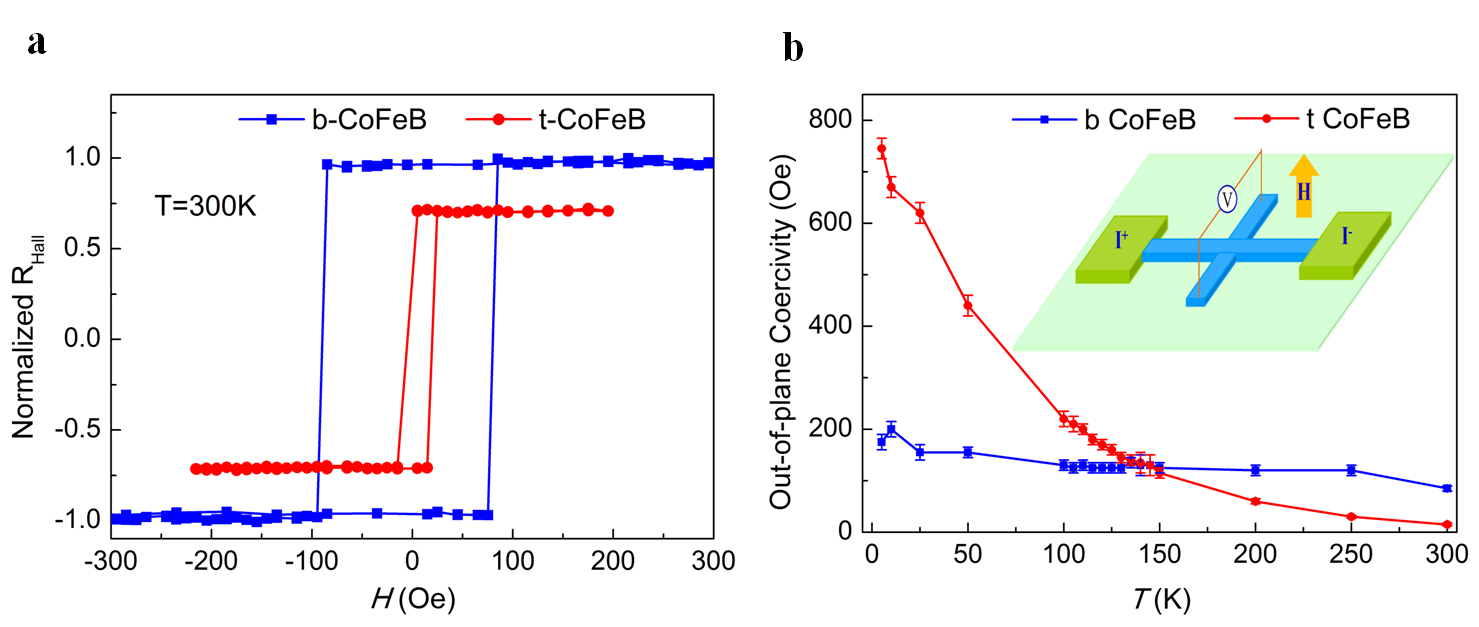


Figure S3. a, Normalized Hall resistance loops of Ta/CoFeB(1.2nm)/MgO and Ta/MgO/ CoFeB(1.4 nm) at RT. b, Temperature dependence of coercivity of the CoFeB layers, obtained from the Hall resistance hysteresis loops. The inset shows the schematics of anomalous Hall resistance measurement setup.

**S4. Model of magnetization switching via domain wall nucleation and domain wall expansion**

Here we provide further details on our model. We define the diameter *L* of the circular reverse domain. The domain wall that separates the reversed and un-reversed domains would have a circumference of π*L*. The magnetic energy for a given *L* is thus,

$\delta E=-2H\frac{\pi L^{2}d}{4a_{0}^{3}}+\varepsilon\frac{\pi Ld}{a_{0}^{2}}$ (2)

where the first term represents the reduction of the Zeeman energy of the reverse domain in the external reverse field *H* and the second term is the domain wall energy. In Eq. (2), *d* is the thickness of the film, $a_{0}$is the unit cell length (lattice constant for simple cubic structure), $\varepsilon=(\pi/2)\sqrt{AK}$is the domain wall energy per area with *A* exchange stiffness and *K* anisotropy constant. Minimizing the energy with respect to L, we obtain the energy barrier,

$\delta E_{b}=\frac{\pi\varepsilon^{2}d}{2Ha_{0}}$ (3)

And the critical size of the reverse domain is

$L=L_{b}\equiv a_{0}\frac{\varepsilon}{H}$ (4)

At finite temperature, the magnetization switching is taking place via thermal activation in which the reverse domains are nucleated. The reverse domains are expanded under thermal agitation. When the domain reaches a critical size of Eq.(4), the spontaneous switching follows. The time needed to reverse the magnetization (relaxation time) is given by,

$\int_{0}^{t_{m}} dt\exp\left(-\frac{\delta E_{b}}{k_{B}T}\right)=f_{0}^{-1}$ (5)

Where $t_{m}$is the measured time, $f_{0}$ the attempt frequency, and *T* the temperature. For a time independent energy barrier, Eq.(5) reduces to the conventional Neel-Brown relaxation time.

The reversal probability P at the time interval *dt* for overcoming the energy barrier, Eq.(2) is given by Neel-Brown’s thermal agitation equation:

$dP=(1-P)f_{0}dt\exp[-\delta E_{b}/k_{B}T]$ (6)

The above equation leads to,

$-\ln(1-P)=f_{0}\int_{0}^{t_{m}} dt\exp\left[-\left(\frac{\pi\varepsilon^{2}d}{2Ha_{0}k_{B}T}\right)\right]$ (7)

Where $t_{m}$is the experimental measuring time. If a constant reverse magnetic field is applied, the coercivity can be readily derived from the above equation by setting$P=1-e^{-1}$**,**

$H=H_{c}=\frac{\Delta(T)}{[\ln(f_{0}t_{m})]}$ (8)

where$\Delta(T)=\pi\varepsilon^{2}d/2a_{0}\kappa_{B}T$.$\varepsilon=(\pi/2)\sqrt{AK}$is the domain wall energy per area with *A* exchange stiffness with *K* anisotropy constant. The fitting parameters are shown in the table 1. If a non-consistent *H* is applied, e.g.,$H=H_{0}\sin(\omega t)$, an approximation is needed to integrate out the time. In the case where ω is much less than the attempt frequency ～GHz, the coercivity remains the same as Eq.(8).

Figure S4 shows the MOKE dynamic hysteresis loops for t- and b-CoFeB films under the alternate magnetic field with different amplitudes and a fixed frequency of 50 Hz at 300 K. The fitting experimental curves and parameters are shown in the Figure S5 and table 1, respectively. For the top CoFeB layer (t-CoFeB), the thermal assisted model fits our measurement data much better at 100 K and 200 K, but not 300K, while for the bottom CoFeB layer (b-CoFeB), the model works very well for all temperatures.


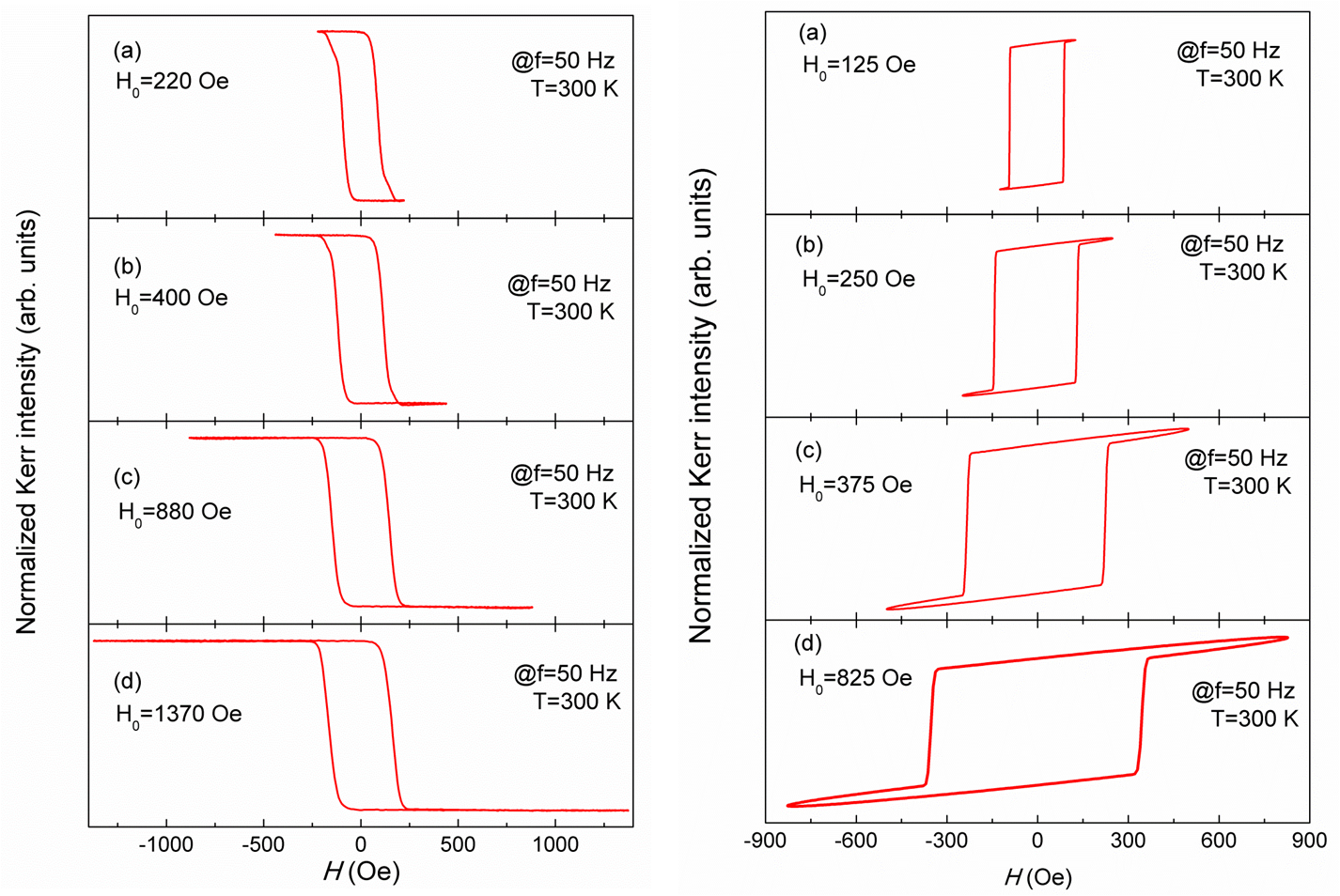


Figure S4. The MOKE hysteresis loops for t- and b-CoFeB film with the alternate magnetic field amplitude with a fixed frequency of 50 Hz at RT.


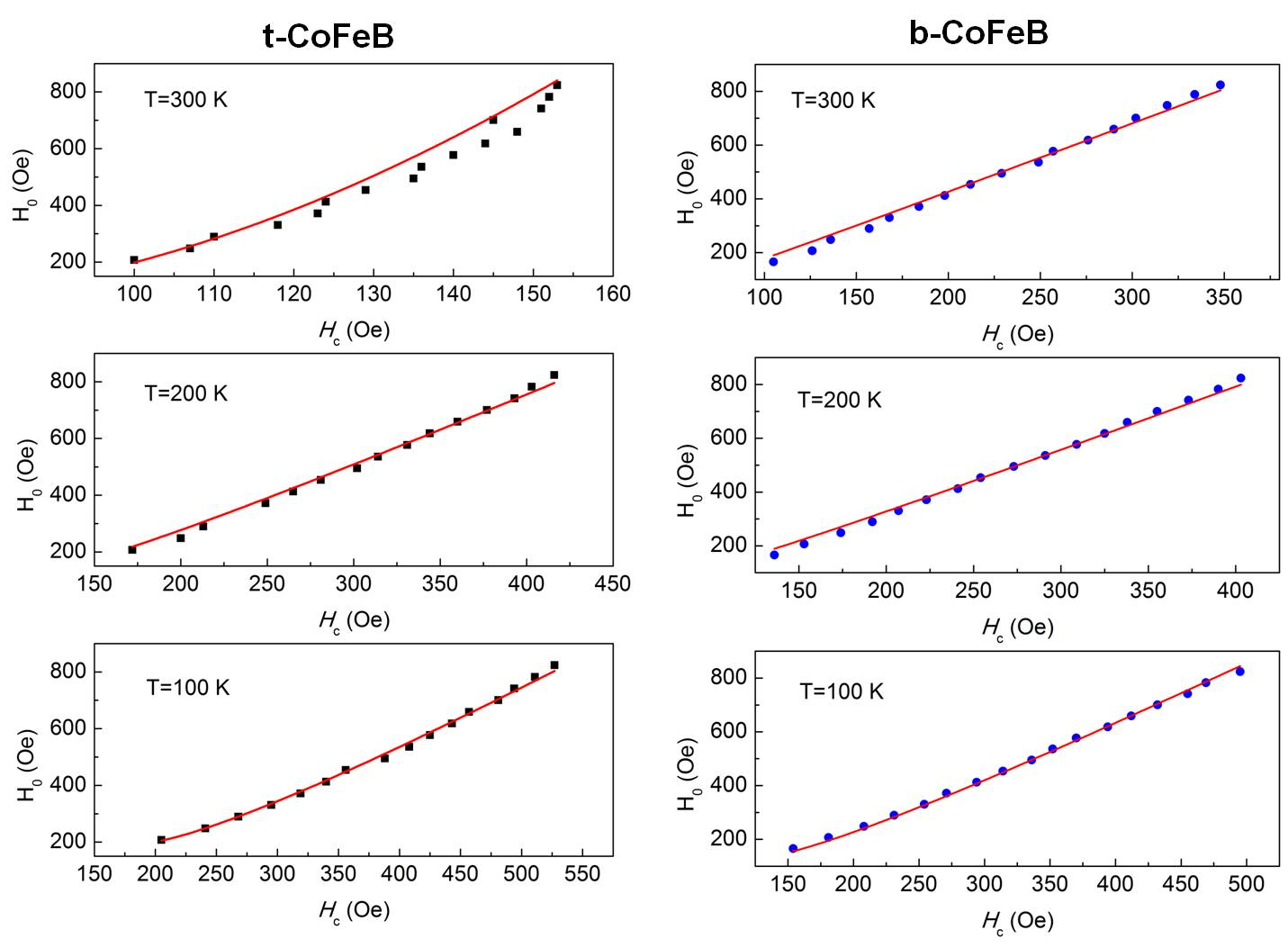


Figure S5. The coercivity of top CoFeB (t-CoFeB) and bottom CoFeB (b-CoFeB) layer as a function of the amplitude of applied magnetic field at 100 K, 200 K and 300 K.

Table 1. The fitting parameters of the dynamic coercivity for t-CoFeB and b-CoFeB film as a function of the magnetic field with alternate amplitude and frequency at 100 K, 200 K and 300 K, according to Eq. (8).

| *T* (K) | ε (J/m^2^) | | *f*_0_ (Hz) | |
| --- | --- | --- | --- | --- |
|  | t-CoFeB | b-CoFeB | t-CoFeB | b-CoFeB |
| 100 | 1.25×10^-2^ | 0.94×10^-2^ | 735 | 728 |
| 200 | 0.92×10^-2^ | 0.64×10^-2^ | 812 | 742 |
| 300 | 1.33×10^-2^ | 0.47×10^-2^ | 11497 | 812 |

**S5. The Joule heat of tunneling current through magnetic tunnel junctions**

When tunneling current passes through the magnetic tunnel junction (MTJ), Joule heat can be generated, resulting in temperature increasing Δ*T*. The temperature distribution *T*(x) inside the MTJ may be modeled by 1d thermal diffusion equation^3^

$C_{v}\frac{\partial T}{\partial t}=\kappa\frac{\partial^{2}T}{\partial x^{2}}+J^{2}/\sigma$ (9)

For steady state condition of$\partial T/\partial t=0$, Eq.(9) is simplified as $\kappa\partial^{2}T/\partial x^{2}+J^{2}/\sigma=0$, where *J, κ* and *σ* are the current density, the thermal and electric conductivities, respectively. The thermal conductance of the MgO barrier can be estimated with $[\kappa_{B}^{-1}+(\kappa_{t}t_{b})^{-1}]^{-1}$ relations^4^. For *t_b_*=2 nm, *κ=0.049 W/m•K*. The average variation of temperature Δ*T* [*T*(x)-T_0_, T_0_=300 K] occurs essentially in the MgO barrier, owning to the lowest value of electric conductance during the stack layers in the MTJ. When the bias voltage *V*_bias_=0.4 V applied on the MTJ, the average variation temperature ΔT is about 280 mK.

**Reference**

1. Hurd, C. M. The Hall effect and its application, edited by C. L. Chien and C. R. Westgate (New York: Plenum Press, 1980).
2. Oiwa, A. *et al*. Magnetic and transport properties of the ferromagnetic semiconductor heterostructures (In,Mn)As/(Ga,Al)Sb, *Phys. Rev.* B **59**, 5826 (1999).
3. Zhang, Z. H. *et al*. Seebeck rectification enable by intrinsic thermalelectrical coupling in magnetic tunneling junctions, *Phys. Rev. Lett.* **109**, 037206(2012).
4. Lee, S. & Cahill, D. Heat transport in thin dielectric films, *Microscale thermophys. Eng*. **1**, 47 (1997).
